# CXCL12 impact on glioblastoma cells behaviors under dynamic culture conditions: Insights for developing new therapeutic approaches

**DOI:** 10.1371/journal.pone.0315038

**Published:** 2024-12-23

**Authors:** Wiam El Kheir, Sahar Naasri, Bernard Marcos, Nick Virgilio, Benoit Paquette, Nathalie Faucheux, Marc-Antoine Lauzon

**Affiliations:** 1 Faculty of Engineering, Department of Chemical Engineering and Biotechnological Engineering, 3D Dynamic Cell Culture Systems Laboratory, Université de Sherbrooke, Sherbrooke, QC, Canada; 2 Faculty of Engineering, Department of Chemical Engineering and Biotechnological Engineering, Laboratory of Cell-Biomaterial Biohybrid Systems, Université de Sherbrooke, Sherbrooke, QC, Canada; 3 Faculty of Medicine and Health Sciences, Department of Medical Imaging and Radiation Sciences, Université de Sherbrooke, Sherbrooke, QC, Canada; 4 Faculty of Engineering, Department of Chemical Engineering and Biotechnological Engineering, Université de Sherbrooke, Sherbrooke, QC, Canada; 5 Department of Chemical Engineering, Polytechnique Montréal, Montreal, QC, Canada; 6 Clinical Research Center of the Centre Hospitalier Universitaire de l’Université de Sherbrooke, Sherbrooke, QC, Canada; 7 The Quebec Network for Research on Protein Function, Engineering and Applications, Montreal, QC, Canada; 8 Research Center on Aging, Sherbrooke, QC, Canada; Hong Kong Metropolitan University, HONG KONG

## Abstract

Glioblastoma multiforme (GBM) is the most prevalent malignant brain tumor, with an average survival time of 14 to 20 months. Its capacity to invade brain parenchyma leads to the failure of conventional treatments and subsequent tumor recurrence. Recent studies have explored new therapeutic strategies using a chemoattracting gradient to attract GBM cells into a soft hydrogel trap where they can be exposed to higher doses of radiation or chemotherapy. It has been demonstrated *in vitro* under static conditions, that nanoparticles (NPs) encapsulating the chemoattractant CXCL12 can create a gradient to attract GBM cell. However, GBM cell invasion is also largely dependent on interstitial fluid flow (IFF). In the present study, a custom-made *in vitro* 3D model with indirect perfusion to mimic IFF at flow rates of 0.5 μL/min and 3 μL/min was used to examine the invasive behavior of F98-rodent-derived and U87-human-derived GBM cells. This model simulated IFF and CXCL12 gradient within an alginate:matrigel-based hydrogel mimicking brain parenchyma. Findings revealed that CXCL12 (1600 ng/mL) released from NPs significantly increased the migration of F98 GBM cells after 72 hours under IFF conditions at both 0.5 and 3 μL/min. In contrast, U87 GBM cells required a higher CXCL12 concentration (2400 ng/mL) and longer incubation time for migration (120 hours). Unlike the F98 cells, U87 GBM cells showed a CXCL12 dose-dependent proliferation. Semi-quantitative qPCR showed higher CXCR4 mRNA levels in F98 cells than in U87 cells. CXCL12 significantly increased intracellular calcium levels via CXCR4 activation, with a 2.3-fold rise in F98 cells compared to U87, consistent with observed cell behavior during perfusion. This highlights the combined influence of IFF and CXCL12 on cell migration, dependent on cell line. This 3D dynamic model is a valuable tool to analyze parameters like interstitial fluid flow (IFF) and chemokine gradients, influenced by GBM tumor diversity.

## Introduction

Glioblastoma multiforme (GBM) is a grade IV primary brain tumor. It represents the most aggressive form of all malignant brain cancer accounting for half of them [1]. The standard treatment for this malignant neoplasm consists in a surgical resection of the tumor to reduce its burden followed by concomitant adjuvant radio-chemotherapy treatments [2]. Albeit the fierceness of the treatment, after age 40, the survival rate at one and five years is around 41% and 6%, respectively [1] mainly because of the tumor heterogeneity at both molecular and cellular levels [3], and the presence of the blood–brain barrier (BBB) that control the systemic drugs administration [4]. As the standard treatments for GBM remain mostly ineffective to eradicate all cancerous cells including those infiltrated in the parenchyma, the tumor recurrence is inevitable. Therefore, many research groups focused on understanding how GBM tumor cells infiltrate brain tissue [5], and how this mechanism can be halted [6–10]. The migration of the GBM cells is a complex process depending on their interactions with the surrounding microenvironment [11,12], fluids dynamics such as the interstitial fluid flow (IFF), and molecules such as chemoattractants [9]. Instead of trying to eliminate GBM cells infiltrated in the brain, we propose to inverse the direction of GBM cell migration towards a well confined area (a soft hydrogel-based trap) in which they will be trapped and can be eliminated with localized radiotherapy using a chemoattractant gradient. The device proposed takes the name of gliotrap (GBM-trapping), and it combines an alginate macroporous hydrogel functionalized with RGD peptides (for cells catching) and alginate–chitosan composite NPs encapsulating chemokine (for cells attraction) [13]. The hydrogel will be loaded by alginate–chitosan NPs and implanted into the surgical cavity of the tumor after its resection to ensure a chemokine gradient maintained for a very long period.

Among several chemoattractants and their receptors, CXC chemokine ligand 12 (CXCL12) also known as stromal-cell derived factor 1 (SDF-1) and its receptor CXC type 4 (CXCR4) are involved in the proliferation and migration of GBM cells. The chemokine and its receptor have been investigated to reverse the migration of GBM cells to trap them within one confined area to facilitate their removal [14,15]. Further, it has been shown that the IFF also plays a critical role in the GBM cell invasion of the brain parenchyma by enhancing chemokines secretion along white matter tracts [16–18].

The IFF can be defined as the movement of fluid between cells in the interstitial space, mainly composed of water, ions, and gaseous and organic molecules (O_2_, CO_2_, hormones) [19]. It develops due to the high interstitial pressure between the tumor and the healthy tissue [20]. Different techniques are used to measure the interstitial fluid flow such as MRI and intravital microscopy. IFF was found to be 0.61 mm/h in the white matter tracts of the temporal lobe of rats [17], whereas bulk flow rates of 0.1–0.3 μL/min were reported in rat brains along the perivascular spaces and axon tracts (in rats) [21]. Tumor’s progression is marked by an increase in the IFF that plays a critical role in GBM cells migration through increasing their expression of CXCR4 and secretion of CXCL12 along white matter tracts, blood vessels, and subpial regions [6,16].

Three-dimensional (3D) *in vitro* dynamic models that mimic different elements of the *in vivo* cancerous cell microenvironment are better to develop new therapeutic approaches as they also allow cells to express their aggressive and metastatic potential [6,10]. Cavo et al. demonstrated that breast cancer cells (MDA-MB-231) can migrate under a 3D *in vitro* dynamic culture system that mimics the peritumoral space by using an hydrogel made of Alginate:Matrigel 50:50 (Alg:M) and a bioreactor-based invasion assay [22]. In addition, the cancerous cells seeded in the Alg:M can grow *in vitro* while expressing their *in vivo* characteristics, especially invadopodia formation [22]. However, this 3D dynamic culture model lacked control over the flow rate, which is an important parameter that may influence cell migration.

GBM tumors may be in different brain areas, some more irrigated than others which implies the presence of different IFF. The control of the flow rate in 3D dynamic model is particularly relevant, especially considering the application of CXCL12, as a mean to attract GBM cells that have invaded the surrounding peri-tumoral tissue post-tumor resection. A critical aspect of this approach is the characterization of the required chemokine dosage needed to establish an adequate gradient for effective cell migration.

We have developed in our previous work a 3D *in vitro* model that takes into consideration the advective contribution of the IFF to study CXCL12 release and distribution under conditions that mimic the human brain environment [13]. The model consists of a perfusion bioreactor system that offers many advantages, such as a precise control of the flow (0.5, 3, 6.5 and 10 μL/min) under physiological-like conditions [13]. Our study revealed a significant influence of clinically pertinent indirect perfusion flow rates on CXCL12 release and gradient, both in and against the flow direction. Notably, an increasing flow rate amplified the effect of advection over diffusion, thus increasing drastically the CXCL12 release rate. For example, the use of flow rates at 6.5 μL/min and 10 μL/min enabled the release of a significant portion of the initial chemoattractant mass loading within the collected media, while only a small quantity of CXCL12 remained entrapped within the hydrogel [13]. Whereas the use of 0.5 μL/min and 3 μL/min that reflect poorly irrigated brain zones effectively stimulated the diffusive conditions necessary in the brain parenchyma for creating a concentration gradient that can attracts GBM cells [13]. Therefore, based on our previous research, experiments were conducted at 0.5 μL/min and 3 μL/min flow rates in the present study.

Using this unique 3D dynamic perfusion system [13], we investigated the behaviors of two GBM cell lines (rodent-derived F98-mCherry and human-derived U87-GFP) in an Alg:M hydrogel. We have been able to track the cells migration over time and, subsequently, we demonstrated that different cell types respond differently to the same flow rates and CXCL12 concentration gradient. The model we have developed allowed the study of the migratory behavior of various cancer cells under conditions that more accurately mimic the fluid dynamics found in the brain. This represents a versatile model of high interest for the development of future therapeutic strategies.

## Materials and methods

### Materials

PRONOVA® UP LVG medical grade sodium alginate with a guluronic acid (G) to mannuronic acid (M) ratio ≥1.5 and apparent viscosity of 20–200 mPa∙s was purchased from NovaMatrix (Sandvika, Norway). High molecular weight (HMW) chitosan (>310 kDa) with > 85% deacetylation and calcium chloride (CaCl_2_) were purchased from Millipore-Sigma (Sigma-Aldrich, Oakville, ON, Canada). Human recombinant CXCL12 (SDF-1α) (purity ≥98%, PeproTech United States, Rocky Hill, NJ, United States) was reconstituted in sterile Milli-Q water following the manufacturer’s instruction. Matrigel^®^ growth factor reduced (GFR) basement membrane matrix was purchased from Corning^®^ (Thermo Fisher Scientific, Canada). Penicillin (10,000 Units/mL)-streptomycin (10,000 μg/mL) antibiotics (Gibco), amphotericin B (250 μg/mL) (Gibco), cell culture medium DMEM (with red phenol, high glucose, GlutaMAX™ Supplement, pyruvate 1X) (Gibco) and Hoechst 33342 staining solution were purchased from Life Technologies (Burlington, ON, Canada). Fetal bovine serum was purchased from Wisent Bioproducts (St-Bruno, QC, Canada). DNeasy^®^ Blood and tissue kit was purchased from Qiagen (Toronto, ON, Canada). Polydimethylsiloxane (PDMS) (Sylgard^TM^ 84, Dow Chemical) was purchased from Ellsworth Adhesives Canada (Stoney Creek, ON, Canada).

### Methods

#### Nanoparticles synthesis

Alginate/Chitosan-Nanoparticles (Alg/Chit-NPs) were synthesized using the ionotropic gelation method as previously described by Gascon et al., [23]. Briefly, under aseptic conditions, CXCL12 was added to an alginate solution (1 mg/mL) to achieve a final concentration of 1600 ng/mL and left under agitation for 5 min. Calcium chloride solution (18 mM) was then added dropwise to the alginate solution for a final Alg:CaCl_2_ ratio of 5:1 (w/w) under constant and vigorous stirring to promote electrostatic interactions. After 30 min, high molecular weight chitosan solution (1 mg/mL in 1% (v/v) glacial acetic acid) was added dropwise to the alginate/CaCl_2_ solution for a final ratio of 4:1 mass to mass. The mixture was left overnight under vigorous agitation at 4°C. The Alg/Chit-NPs solution was then centrifuged at 20,000 × g for 30 min at 10°C. The supernatant was eliminated, and the pellet was resuspended in sterilized Mili-Q water for a final concentration of 10 mg/mL. However, as previously described in El Kheir et al. [13], the Alg/Chit NPs retain 40% of CXCL12, therefore, the Alg/Chit-NPs CXCL12 solution was increased by 40% in order to obtain a final concentration of 1600 ng/mL or 2400 ng/mL in the hydrogels [13].

#### Alginate 1% and alginate: Matrigel hydrogel preparation

*Alginate 1% hydrogel*. The preparation of Alg 1% (w/v) and Alg:M hydrogels were previously described in El Kheir et al., [13]. Briefly, medical-grade sodium alginate (NovaMatrix) was mixed with sterilized Milli-Q water or DMEM 1× (supplemented with 0.1% (w/v) bovine serum albumin purchased from Millipore-Sigma) for 12 h under vigorous stirring to achieve a final concentration of 1.5% (w/v). The solution was filtered using a 0.45 μm filter and stored at 4˚C. CXCL12 solution was mixed with alginate solution and water to achieve a final concentration of 1% (w/v). A solution of CaCl_2_ (18 mM) was then added to the mixture for gelation. The gel was left for 30 min at room temperature to ensure proper gelation.

*Alg*:*M (50*:*50) hydrogel*. Alg:M (50:50) hydrogel offers to cancer cells an adequate environment to express their malignant behaviors [22]. As previously described in Cavo et al., Matrigel^TM^ (Corning) was thawed overnight at 4°C on ice prior the hydrogel preparation [22]. Culture chambers were moved into ice 20 min before forming the gels to adapt the matrigel temperature. A final ratio of 1:1 (w/w) alginate and matrigel was prepared using DMEM 1× (Multicell). The chambers were then moved out from ice to add the CaCl_2_ (18 mM) with a final concentration of 1% (w/v) for gelation. The gels were left 30 min at room temperature.

#### Cell culture

The murine F98 GBM cell line was obtained from the American Type Culture Collection (Manassas, VA). Cells were cultured in T75 cell culture-treated polystyrene flasks (Corning, USA) using DMEM medium supplemented with 1% (v/v) penicillin (10,000 U/mL), streptomycin (10,000 μg/mL), amphotericin B (250 μg/mL) and 10% (v/v) heat inactivated fetal bovine serum (FBS). Cells were grown in a humidified environment at 37°C with 5% CO_2_ and 95% air. Every 3 days, cells were passed into fresh culture medium. The mCherry fluorescent marker was introduced into the F98 cells using the pCDH-CMV-mCherry-T2A-Puro lentiviral vector (Addgene Catalog#72264, Watertown, MA), as previously described in Bouchard et al., [24].

The gene encoding the GFP protein was introduced into the U87 GBM cells to enable their detection and assessment through fluorescence imaging. The U87-GFP cell line was generated by lentiviral infection of a GFP/pLenti6/V5 construct. More specifically, human 293T cell line was transfected with the plasmids pLP1, pLP2, pLP/VSV-G, and the lentiviral plasmid GFP/pLenti6/V5. After 48 h of incubation, the supernatant containing the lentivirus was harvested and filtered through a 0.45 μm filter. Human U87 GBM cells were infected with the virus in the presence of 4 μg/mL of polybrene. After 48 h of incubation, the virus solution was replaced with culture medium containing blasticidin as a selection agent, and the cells were incubated for 10 days. Cells that have integrated the GFP/pLenti6/V5 vector are resistant to blasticidin. U87-GFP cells were grown in a humidified environment at 37°C with 5% CO_2_ and 95% air on T75 tissue culture flasks in DMEM medium supplemented with 1% (v/v) penicillin (10,000 U/mL), streptomycin (10,000 μg/mL), amphotericin B (250 μg/mL) and 10% (v/v) FBS. Every 3 days, cells were passed into fresh culture medium.

#### Cell migration assay

**Fig 1:**
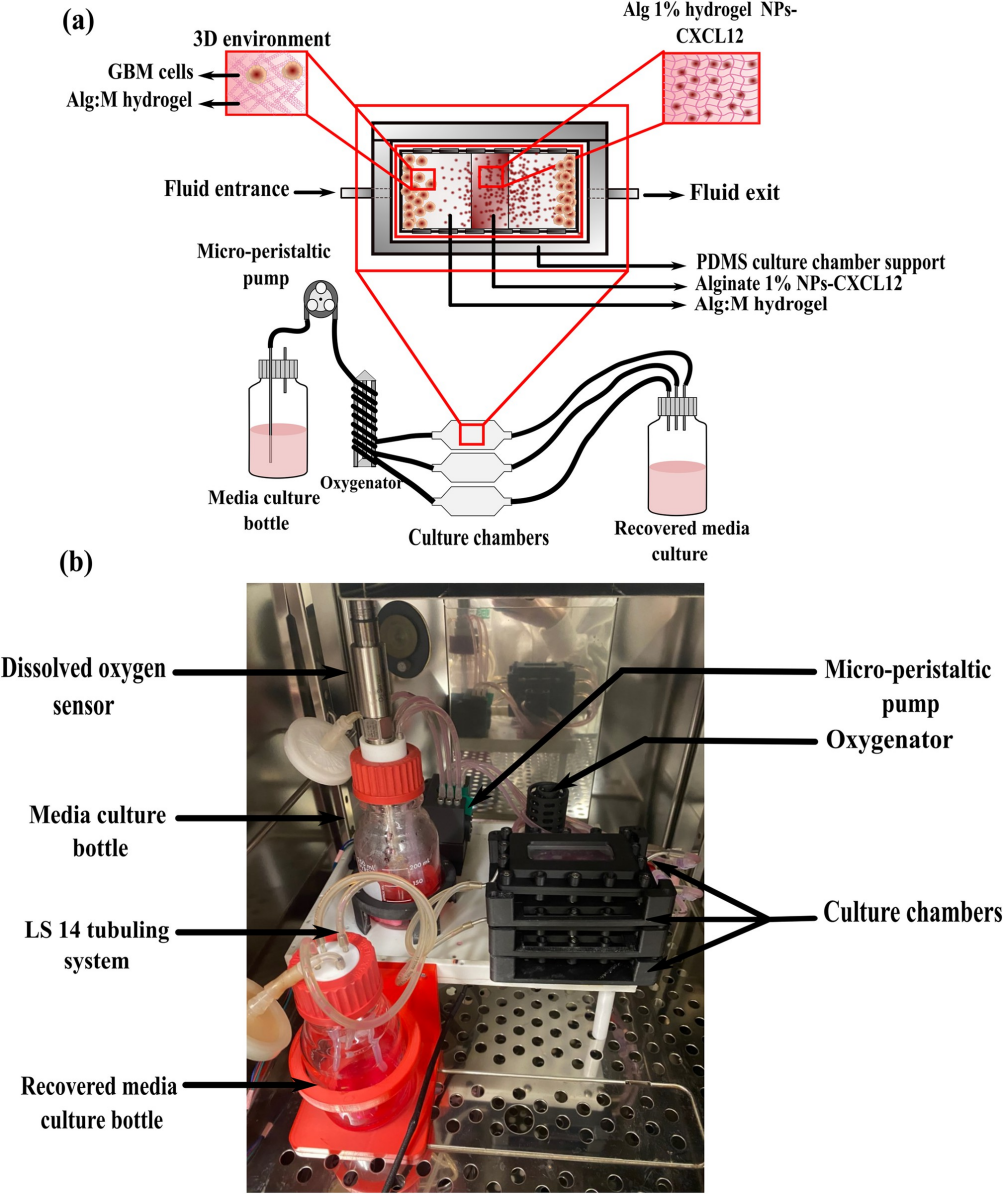
Different steps for gels preparation. GBM: Glioblastoma multiforme; Alg 1%: Alginate hydrogel 1%; Alg/Chit-NPs CXCL12: Alginate/Chitosan-Nanoparticles loaded with CXCL12; Alg:M: Alginate:Matrigel (50:50); CXCL12: C-X-C motif chemokine 12.

*Gels preparation*. The preparation of the gels for cell migration involves four major steps. Each step serves to mold a section of the gel as illustrated in **Fig 1**.

Step1: Molding of Alg 1% hydrogel containing Alg/Chit NPs-CXCL12

The first step consists in molding the central section of the Alg1% hydrogel containing Alg/Chit-NPs loaded with CXCL12. Using mold support separators, culture chambers were positioned on ice and left for 5 min to adapt to the matrigel temperature. At the center, alginate 1% (w/v) hydrogel containing Alg/Chit-NPs loaded with CXCL12 was formed by a successive deposition of two layers with same gel volume to ensure an optimal gelation and a uniformly distributed Alg/Chit-NPs-CXCL12.

Each layer requires approximately 15 min for gelation. Subsequently, the separators were gradually removed from the middle towards the extremities to allow a seamless integration of each gel section, as illustrated in **Fig 1**.

Step 2: Molding of Alg:M hydrogel sections

Afterwards, the second step serve to seed the Alg:M hydrogels in the two compartments next to the Alg 1% hydrogel. Alg:M hydrogels were jellified by continuous deposition of four identical layers (same gel volume). Each layer was left for 15 min for gelation and 40 min at the end. Then, the separators were removed, and the chambers were taken out from ice to allow gelation of the gels (**Fig 1**).

Step 3: Molding of Alg:M hydrogel sections containing mCherry-F98 or GFP-U87 GBM cells

Subsequently, the third step of the preparation consist of seeding Alg:M hydrogels containing mCherry-F98 or GFP-U87 GBM cells in the extremities. The mixtures comprising matrigel, CaCl_2_, and either F98-mCherry or U87-GFP cells (at approximately 1.5 × 10^6^ cells/mL) were prepared. The suspension was briefly vortexed for 20 seconds to ensure uniform distribution of the cells within the suspension. Alg 1% (w/v) solution containing DMEM 1× with red phenol was combined with the cell suspension, and the resulting mixture was promptly added to the culture chambers. The mixture was left undisturbed for 30 to 40 min to facilitate gelation.

Step 4: Culture chambers connection to the perfusion bioreactor

Subsequently, the mold supports were removed from the culture chambers. Following gelation, the chambers were affixed within the bioreactor using culture chamber supports to ensure complete sealing and the integrity of the system (**Fig 1**).

**Fig 2:**
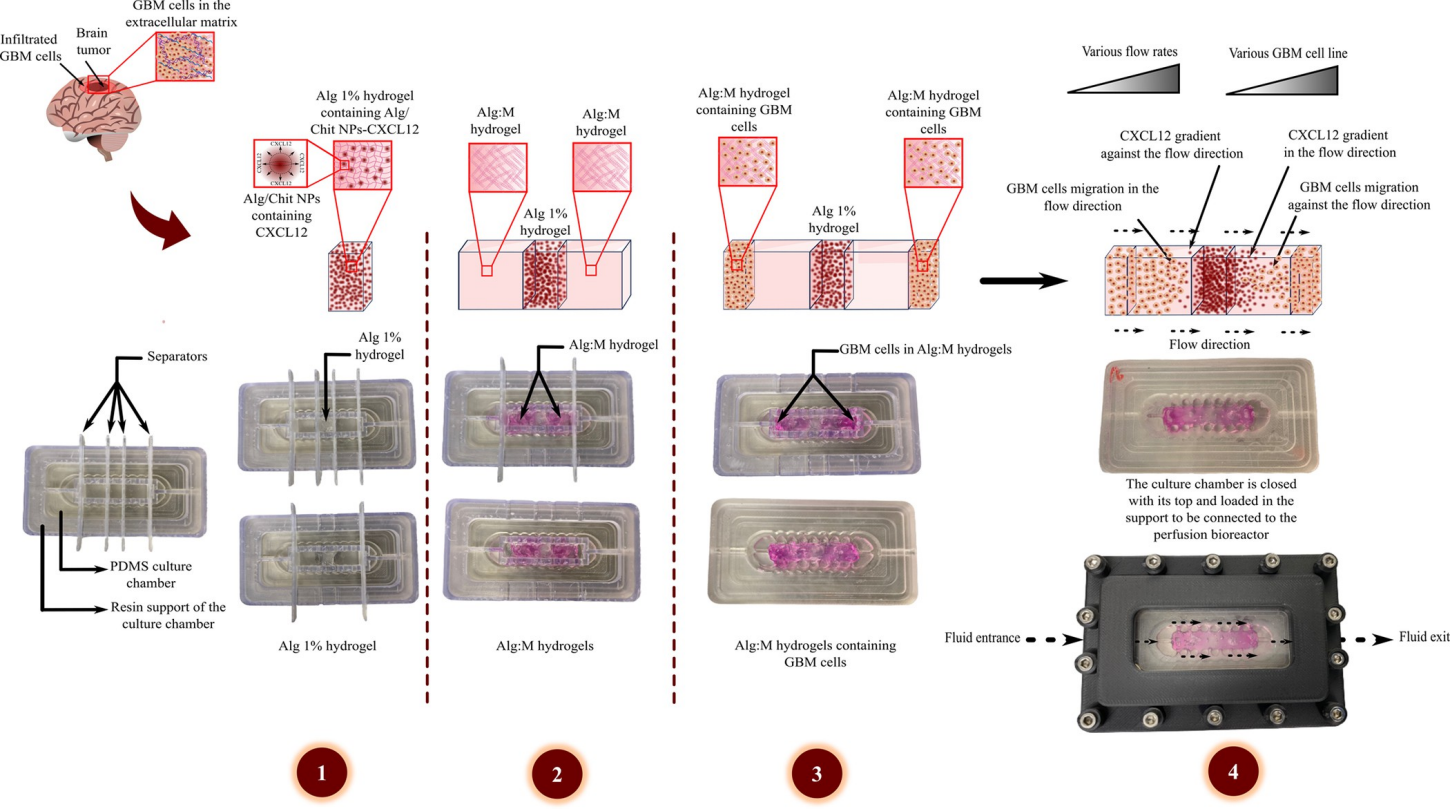
(a) Schematic representation of the perfusion bioreactor system for GBM cells migration assay. (b) The perfusion bioreactor assembly. GBM: Glioblastoma multiforme; Alg 1%: Alginate hydrogel 1%; Alg/Chit NPs-CXCL12: Alginate/Chitosan-Nanoparticles loaded with CXCL12; Alg:M: Alginate:Matrigel (50:50); PDMS: Polydimethylsiloxane; 3D: three-dimensional.

*Perfusion bioreactor preparation*. As previously described in El Kheir et al. [13], we developed a perfusion bioreactor that simulates the brain IFF (**Fig 2**). The device was engineered to deliver dynamic continuous indirect perfusion to a 3D scaffold. It also provides enhanced insights into the influence of flow dynamics on the release of chemokines within an *in vitro* 3D environment. The system allowed the control of several conditions including temperature, CO_2_, dissolved O_2_ levels (dO_2_), perfusion flow rate, and hydrogel composition. The operational principles of the perfusion bioreactor have been discussed in our previous work [13]. Briefly, a gas permeable platinum-cured silicone tubing system (Masterflex®) facilitated the delivery of supplemented DMEM containing 1% (v/v) antibiotics from the media bottle to the culture chambers, perfused by a custom-designed micro-peristaltic pump as illustrated in **Fig 2**. The flow rate is proportional to the rotational speed of the pump rotor as the peristaltic pump is a positive-displacement system. Meanwhile, in the present study, the media culture is collected in a recovery bottle.

*Fluorescence scans*. Supported by a custom-made holder, epifluorescence scans of the entire culture chambers, were performed at t = 0 h, t = 24 h and t = 72 h (and t = 120 h for U87-GFP) using the EVOS™ FL Auto Imaging System (Life Technologies) with a magnification of 4× for the scans and ×10 for the individual images (zoomed sections). The RFP light cube was used for F98-mCherry GBM cells, whereas the GFP light cube was used for U98-GFP cells and the DAPI light cube for nucleus (Hoechst) staining. These scans were taken without disrupting the ongoing migration assay. Additionally, dO_2_ saturation levels were monitored from the initiation to the conclusion of the experiment, to confirm the system can maintain a high dO_2_ saturation level with measured values ranging between approximately 97% and 98% throughout the duration of the observation period (t = 0 h to t = 72 h or 120 h).

*Cell nuclei staining*. Culture chambers were removed from the bioreactor support after the end of the experiment. Cells nuclei were stained with Hoechst 33342 (5 μg/mL) for 40 min using a plate stirrer to keep the chambers under agitation. Epifluorescence scans were taken for each condition by EVOS^TM^ FL-Auto Imaging System (Life Technologies) as previously described with a magnification 4× using DAPI–RFP light cube for F98-mCherry GBM after 72 h cells and DAPI–GFP light cube for U87-GFP GBM cells after 120 h.

#### DNA extraction and quantification

*Gels dissociation*. At the end of the experiment, the distinct sections of the gels (**a.** Alg:M in the flow direction containing F98-mCherry GBM cells, **b.** Alg:M in the flow direction, **c.** Alg 1% hydrogel containing Alg/Chit-NPs loaded with CXCL12, **d.** Alg:M in the opposite flow direction and **e.** Alg:M in the opposite flow direction containing F98-mCherry GBM cells) were carefully separated for the quantification of the DNA within each segment. The different hydrogels were dissociated using a dissociation solution composed of 16.1 mg/mL sodium citrate, 8.7 mg/mL NaCl, and 10.2 mg/mL ethylenediaminetetraacetic acid (EDTA) with a pH = 7.4. The different sections were transferred into 1.5 mL ultra-low adhesion microtubes, and the dissociation solution was added to each tube. Subsequently, tubes were left for approximately 40 min under medium stirring and the DNA extraction was performed using Qiagen DNeasy Blood and tissue kit (Qiagen) following the manufacturer’s instructions. Briefly, samples were centrifuged for 5 min at 300 × g, resuspended in PBS and lysed using the proteinase K. Samples were then incubated and transferred to a DNeasy Mini spin column. This was succeeded by a series of wash steps. The spin columns were transferred to new 1.5 mL ultra-low adhesion microtubes and DNA was eluted using AE buffer twice.

Quantification of the extracted DNA was performed by measuring the UV absorbance at 260 nm using a microplate reader (Safire2, Tecan US Inc., Morrisville, NC, United States). The concentration of DNA in each hydrogel segment was determined following the Beer- Lambert-law:

$$\mathrm{Log}(\frac{I_{0}}{I})=A=\varepsilon lc$$ (Eq 1)

In which *A* is the absorbance, *ε* is the molar attenuation coefficient or absorptivity of DNA $(\frac{1}{50}){\mu g/mL}^{-1}{cm}^{-1}$, *ℓ* is the optical path length of the well (0.1 cm) and *c* is the concentration of the DNA (information and instruction from Qiagen). DNA quantification results were presented as percentages of DNA concentration at the end of each experiment, compared to the initial concentration at t = 0 h.

*Evaluation of CXCR4 receptor expression by semi-quantitative qPCR*. F98 and U87 GBM cells were seeded in 12-well plates (2 x 10^4^ cells/well) using DMEM culture medium supplemented with 10% FBS and 1% penicillin/streptomycin at 5% CO_2_ and 37°C. Total RNA extractions were performed on cell pellets with the Absolutely RNA Microprep Kit (Stratagene, La Jolla, CA, USA) as recommended by the manufacturer. RNA integrity was assessed with an Agilent 2100 Bioanalyzer (Agilent Technologies, Mississauga, ON, Canada). Reverse transcription was performed with 2 μg of total RNA using reverse transcriptase, random hexamers, dNTPs (Roche Diagnostics, Laval, QC, Canada), and 10 units of RNAseOUT (Invitrogen, Burlington, ON, Canada) following the manufacturer’s protocol in a total volume of 20 μL. All forward and reverse primers (Table 1) were individually resuspended in 20–100 μM stock solution with Tris-EDTA buffer and diluted as a primer pair to 1 μM in RNase DNase-free water (IDT, Coralville, Iowa, USA).

Semi-quantitative PCR (qPCR) reactions were performed in 10 μL in 96-well plates on a Realplex2 thermocycler (Eppendorf, Mississauga, ON, Canada) with 5 μL of 2X FastStart Universal SYBR Green Master mix (Roche Diagnostics, Laval, QC, Canada), 10 ng (3 μL) cDNA, and 200 nM final (2 μL) primer pair solutions.

The following cycling conditions were used: 10 min at 95°C; 50 cycles: 15 sec at 95°C, 30 sec at 60°C, 30 sec at 72°C. Relative expression levels were calculated using the qBASE framework1 and the housekeeping genes Mrpl19, Pum1 and Ywhaz for mouse cDNA and PSMC4, PUM1 and TBP for human cDNA. In every qPCR run, a no template control was performed for each primer pair and these were consistently negative.

**Table 1:** Forward and reverse primer sequences used in the qPCR.

	Forward Sequence	Reverse Sequence
**CXCR4**	5’-TAGCCACCGCATCTGGAGAACC-3’	5’-AGCATTTTCTTCACGGAAACAGGGT-3’
**Mrpl19**	5’- AAGGAGAAAAGTACTCCACATTCCAGAG-3’	5’- TGGGTCAGCTGTAGTAACACGA-3’
**Pum1**	5’-TGAGGTGTGCACCATGAAC-3’	5’-CAGAATGTGCTTGCCATAGG-3’
**Ywhaz**	5’- TCCCCAATGCTTCACAAGCAGA-3’	5’- TCTTGTCATCACCAGCGGCAA-3’
**PSMC4**	5’- CCCAGGAGGAGGTGAAGCGG-3’	5’- GGTCGATGGTACTCAGGATGCG-3’
**PUM1**	5’- TGCCAGTCTCTTCCAGCAGCA-3’	5’-TGATTTGGGGTCAAAGGACGTTGG-3’
**TBP**	5’- GCCTTCCACCTTATGCTCAGGG-3’	5’-TGCTCTTCCAAAATAGAGAGACTGTTGG-3’

*Evaluation of CXCR4 receptor activity in F98 and U87 cells*. Activity of CXCR4 receptor was assessed using the Fluo-4 NW calcium Assay Kit (Molecular Probes, Invitrogen, #F36206, CA, US) which relies on the ability of this receptor to increase the intracellular level of Ca^2+^ ions in response to their ligand, the CXCL12.

The F98 and U87 GBM cells (2 x 10^4^ /well) were incubated 96-well black opaque plate with clear bottom (Corning Incorporated Costar, Catalog#3603, NY, USA) for 24 h at 5% CO_2_ and 37°C. After removing the culture medium, 100 μL of dye loading solution supplied with Fluo-4 NW calcium assay kit was added to each well. After a 30 minutes incubation at 37°C, a first fluorescence reading was taken to determine the background level using the plate reader (λ_ex_ = 494 nm, λ_em_ = 516 nm, HT Synergy, Bio-Tek Instrument, Winooski, VT, USA). A second fluorescence reading was performed after the addition of 40 nM AMD11070 (a selective CXCR4 antagonist, Sigma-Aldrich). Finally, a third fluorescence readout was made after addition of 6 nM (52.2 ng/mL) CXCL12 with or without 40 nM (13.98 ng/mL) AMD11070, a CXCR4 antagonist [25].

#### Statistical analysis

Statistical analysis was performed with GraphPad Prism® software (Version 8.0.2). Statistical significance was determined by the Mann-Whitney nonparametric test for DNA quantification results obtained with F98-mCherry. The nonparametric test Kruskal-Wallis followed by Dunn’s multiple comparison post-hoc test was used with U87-GFP DNA quantification results. Regarding the analysis of qPCR results, a Student t-test was used to calculate the p-value. Only differences with p ≤ 0.05 were considered significant.

## Results

### F98 cells migration under 0.5 μL/min

Prior results showed a positive migration response of mCherry-F98 GBM cells to the selected CXCL12 dose (100 ng/mL) [23]. The choice of this concentration was based on the results of our previous research highlighting that an encapsulated percentage of the chemokine remain trapped inside the delivery system at low flow rates of 0.5 μL/min and 3 μL/min [13]. After 86 h and 96 h, CXCL12 was quantified in the Alg:M section in the flow direction at concentration of 50 ng/mL and 140 ng/mL under 0.5 μL/min and 3 μL/min flow rates respectively [13].

#### Fluorescence scans and Hoechst staining

**Fig 3:**
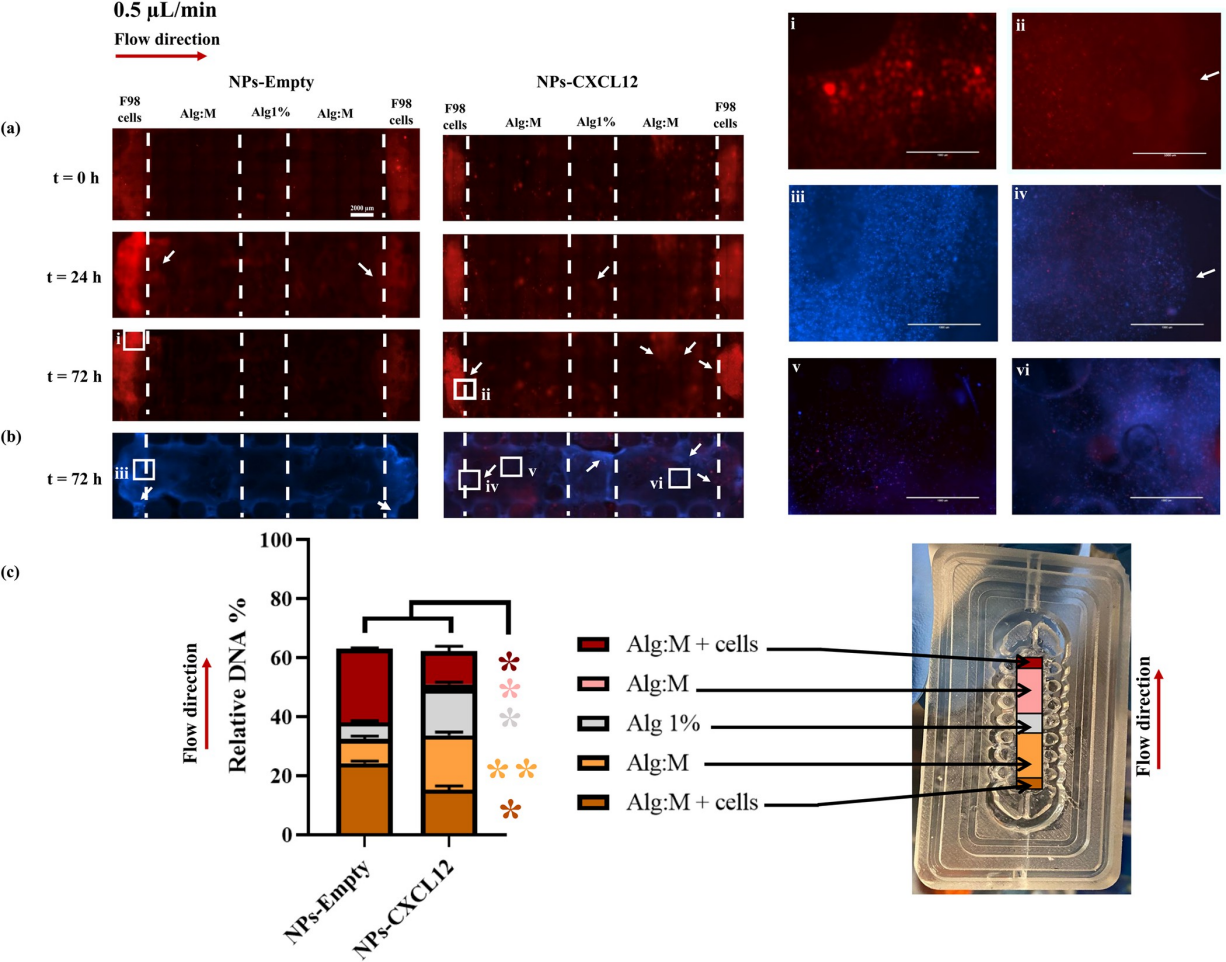
(a) Hydrogels scans of mCherry-F98 GBM cells migration at t = 0 h, t = 24 h and t = 72 h with and without CXCL12 (0.33 μg) under a flow rate of 0.5 μL/min. (b) Hoechst staining of the hydrogels at t = 72h. (c) DNA quantification of the different hydrogel sections at the end of the experiment (N = 15, n = 3). Bars represent the mean ± SD and the squares are zoomed regions. Statistical analysis was performed using a Mann-Whitney t-test, * p ≤ 0.05, ** p ≤ 0.01. The scale bar represents 2000 μm. NPs-Empty: Alginate/Chitosan-Nanoparticles unloaded; NPs-CXCL12: Alginate/Chitosan-Nanoparticles loaded with CXCL12; Alg:M: Alginate:Matrigel (50:50); Alg 1%: Alginate 1% hydrogel.

Culture chambers were connected to the perfusion bioreactor (**Figs 1 and 2**) and filled with the culture medium at 5 rotation per minute. The flow rate was then decreased gradually to reach the targeted perfusion flow rate. mCherry-F98 GBM cells migration was studied under dynamic culture conditions using 0.5 μL/min flow rates with (NPs-CXCL12) and without (NPs-Empty) CXCL12 (**Fig 3**). At t = 0 h, scans showed the localization of F98 GBM cells predominantly at the extremities of each experimental chamber for both experimental conditions, NPs-Empty (control) and NPs-CXCL12, respectively (**Fig 3**A). In contrast, scans at t = 24h revealed a noteworthy difference between the two experimental conditions. Several F98 GBM cells showed migratory behavior toward the Alg:M section of the gel in the direction of flow in the control (NPs-Empty) (**Fig 3**A). However, in the Alg 1% (w/v) hydrogel (central section), only a slight fluorescence was observed in the control, whereas a higher fluorescence intensity was detected in the presence of NPs-CXCL12, showing a higher number of cells reaching the center of the chamber in the presence of CXCL12. Few cells were observed to migrate between 24 h and 72 h in the control (NPs-Empty) (**Fig 3**A; i), while with CXCL12, a cell migration front toward the center of the gel in the direction of the flow was observed at t = 72 h (**Fig 3**A; ii). In the opposite flow direction, in accordance with CXCL12 release (NPs-CXCL12), a marked cell migration was also observed. Nuclei staining of the cells by Hoechst at 72 h confirmed these observation (**Fig 3**B; iii-iv).

#### DNA quantification

The DNA quantification levels for the different hydrogel sections, expressed in percentage as relative DNA amount referred to initial DNA content (relative DNA %), was also assessed with or without CXCL12 (NPs-CXCL12 vs NPs-Empty used as control) to elucidate the impact of the chemokine on the cell distribution (inside or outside the gel) in and against the direction of the flow (**Fig 3**C). Total relative DNA amount was similar at 72 h in both experimental conditions (62.5 ± 1.2% and 62.2 ± 2.8% with and without CXCL12, respectively; p>0.05). This result indicates a similar decrease of ~ 38% in total number of cells for both experimental conditions compared with the initial seeding after 72 h under a flow rate of 0.5 μL/min. These findings were consistent with observations from Hoechst staining scans (**Fig 3**B). In the control (NPs-Empty) at 72 h, the relative DNA amount varied in the different hydrogel sections. DNA quantification revealed that a similar number of cells remained in the Alg:M + cells sections in both the flow directions with a relative DNA amount of 24.3% ± 0.6 and 24.8% ± 0.3 respectively. A relative DNA amount of 8.1% ± 1.0 was quantified in the Alg:M section in the flow direction, whereas no DNA was detected in the Alg:M section against the flow (**Fig 3**C). In the central section Alg 1%, some DNA was detected (relative DNA amount of 5.6 ± 0.5%). In the presence of CXCL12 (NPs-CXCL12), the relative DNA amount at 72 h was significantly decreased in Alg:M + cells sections with the flow direction (15.3% ± 1.12; p ≤ 0.05) and against it (11.2% ± 1.6; p ≤ 0.05) compared with the control (NPs-Empty). In addition, the relative DNA amounts were increased in the Alg:M sections in the direction of the flow (18.4% ± 1.1; p ≤ 0.05) or against it (1.5 ± 0.7; p ≤ 0.01) compared with the control NPs-Empty (**Fig 3**C). Further, a higher relative DNA amount was also detected in the Alg 1% central section of the chamber in the presence of CXCL12 compared with the control (15.6 ± 0.8; p < 0.05). These results underscore the significant influence of CXCL12 under dynamic culture conditions at flow rate of 0.5 μL/min on F98-mCherry GBM cells migratory behavior.

### F98 cells migration under 3 μL/min

#### Fluorescence scans and Hoechst staining

**Fig 4:**
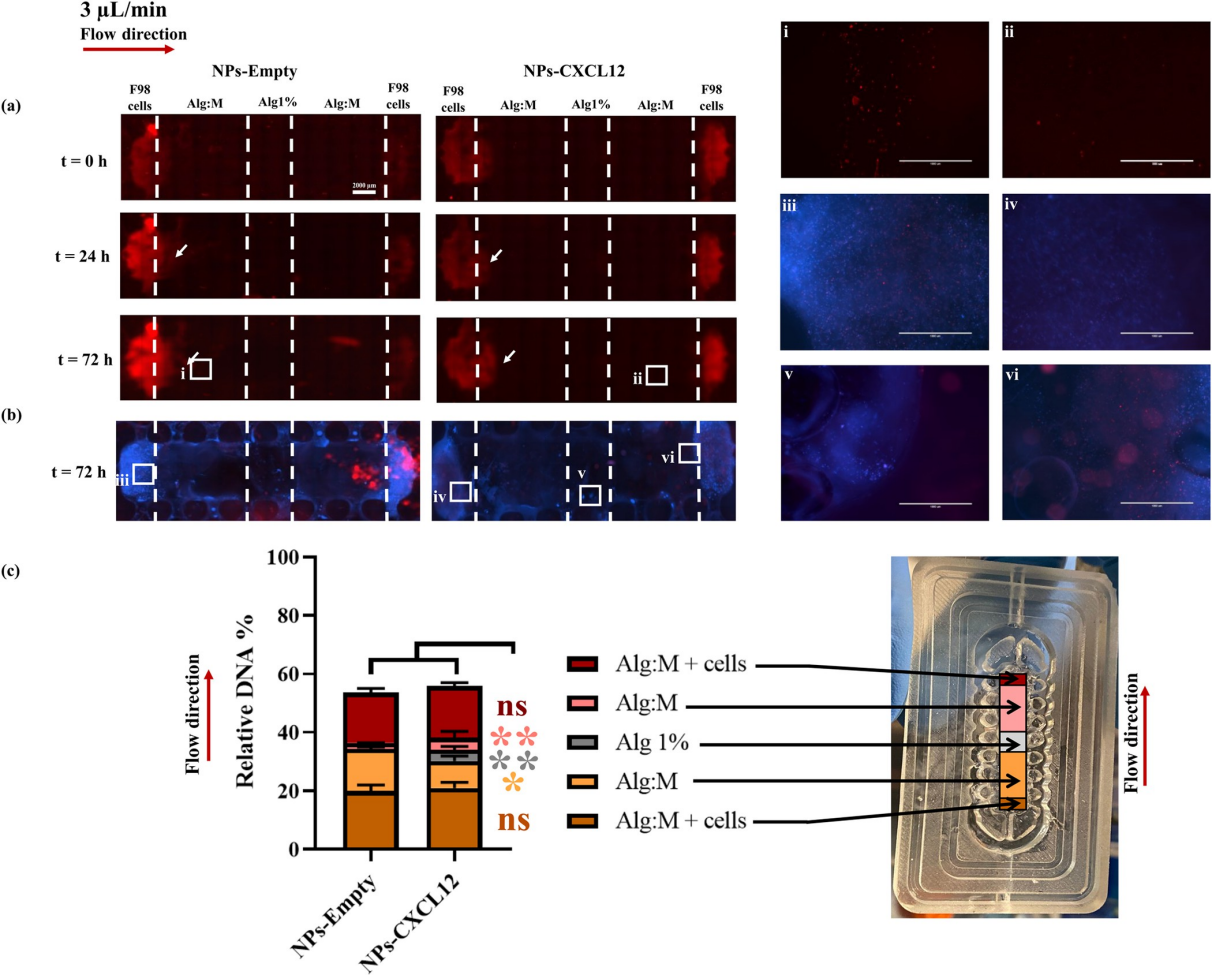
(a) Hydrogels scans of mCherry F98 GBM cells migration at t = 0 h, t = 24 h and t = 72 h with and without CXCL12 at a flow rate of 3 μL/min. (b) Hoechst staining of the hydrogels at t = 72 h. (c) DNA quantification of the different hydrogel sections at the end of the experiment (N = 9, n = 3). Bars represent the mean ± SD and the squares are zoom regions. Statistical analysis was performed using a Mann-Whitney t-test, * p ≤ 0.05, ** p ≤ 0.01. The scale bar represents 2000 μm. NPs-Empty: Alginate/Chitosan-Nanoparticles unloaded; NPs-CXCL12: Alginate/Chitosan-Nanoparticles loaded with CXCL12; Alg:M: Alginate:Matrigel (50:50); Alg 1%: Alginate 1% hydrogel.

Then, mCherry-F98 GBM cells migration was studied with and without CXCL12 under an advective effect obtained by a flow rate of 3 μL/min (**Fig 4**). The scans at t = 0 h indicated the localization of F98 GBM cells before applying the flow for both experimental conditions, control (NPs-Empty) and CXCL12 (NPs-CXCL12) (**Fig 4**A). At t = 24h, mCherry-F98 GBM cells exhibited migration behaviors in the direction of the flow with or without CXCL12, while cell migration against the flow was not clearly observed (**Fig 4**A). At t = 72 h, in both experimental conditions, a significant migration front was observed in the direction of the flow (**Fig 4**A; i). In the presence of CXCL12, few cells were also able to migrate against the flow direction in the Alg:M section (**Fig 4**A; ii). These results were corroborated by Hoechst staining, the migration of cells in the Alg:M sections being promoted in the flow direction and in the Alg 1% (w/v) hydrogel (**Fig 4**B; iii-v). These Hoechst staining scans also confirmed that several cells can be detected in the Alg:M section against the flow in the presence of CXCL12 (**Fig 4**B; vi). The observations were corroborated by the DNA quantification at 72 h (**Fig 4**C).

#### DNA quantification

Total DNA amounts were quite similar in both experimental conditions (NPs-Empty and NPs-CXCL12), and also revealed a reduction of around 40% in DNA level compared to initial amount suggesting a decreased in the cellular number in the chamber after 72 h (**Fig 4**C), as observed in F98 GBM cell and Hoechst staining scans (**Fig 4**A and **4**B). The relative DNA amounts were similar in the Alg:M + cells sections in the flow direction with and without CXCL12 (20.9% ± 2.0 and 19.9% ± 2.1, respectively; p > 0.05). In contrast, the relative DNA amount was increased in the control compared with CXCL12 in the Alg:M section in the flow direction (14.4% ± 0.6 versus 9.3% ± 1.7; p ≤ 0.01). A low amount of DNA was also detected in the Alg:M section against the flow direction in the presence of CXCL12, but it remained higher than that observed with the control (3.9% ± 2.2 NPs-CXCL12 versus 1.9% ± 0.1 NPs-Empty; p ≤ 0.05). While no DNA was detected in the Alg 1% section in the control, 3.9% ± 1.1 were detected in the presence of CXCL12 (p ≤ 0.01 compared to NPs-Empty). These results underscore the significant influence of advective effect on F98-mCherry GBM cells migratory behavior and impact of CXCL12 gradient.

### U87 cells migration under 0.5 μL/min

**Fig 5:**
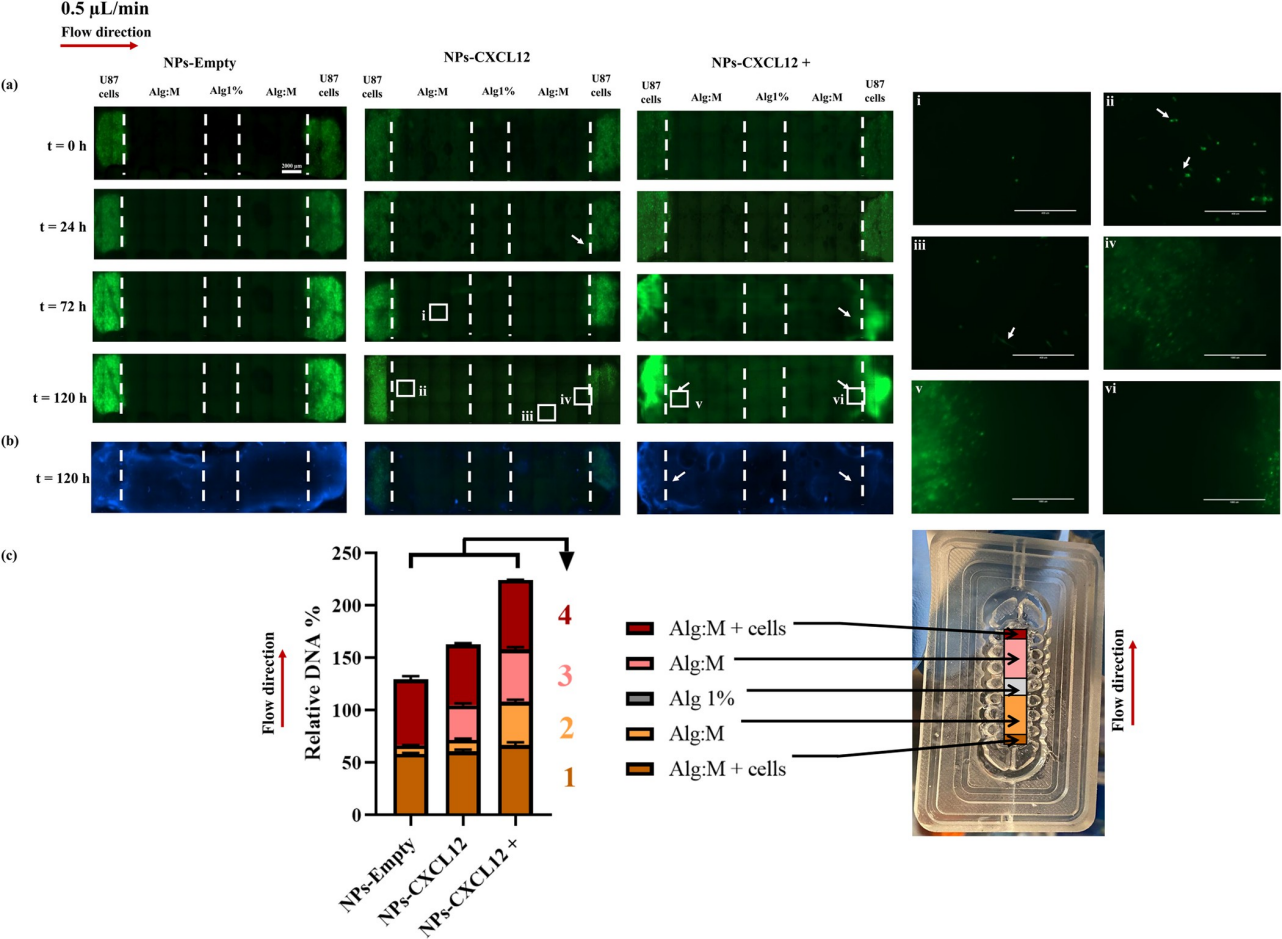
(a) Hydrogels scans of U87-GFP GBM cells migration at t = 0 h, t = 24 h and t = 120 h with and without CXCL12 at a flow rate of 0.5 μL/min. (b) Hoechst staining of the hydrogels at t = 120 h. (c) DNA quantification of the different hydrogel sections at the end of the experiment (N = 9, n = 3). Bars represent the mean ± SD and the squares are zoom regions. Statistical analysis was conducted using a Kruskal-Wallis test followed by Dunn’s multiple comparison post-hoc test, * p *≤* 0.05, ** p *≤* 0.01, *** p *≤* 0.001. **1**: (Alg:M + cells NPs-Empty vs. Alg:M + cells NPs-CXCL12) *, (Alg:M + cells NPs-Empty vs. Alg:M + cells NPs-CXCL12+) *, (Alg:M + cells NPs-CXCL12 vs. Alg:M + cells NPs-CXCL12+) ns. **2**: (Alg:M NPs-Empty vs. Alg:M NPs-CXCL12) ns, (Alg:M NPs-Empty vs. Alg:M NPs-CXCL12+) **, (Alg:M NPs-CXCL12 vs. Alg:M NPs-CXCL12+) ns. **3**: (Alg:M NPs-Empty vs. Alg:M NPs-CXCL12) ns, (Alg:M NPs-Empty vs. Alg:M NPs-CXCL12+) ***, (Alg:M NPs-CXCL12 vs. Alg:M NPs-CXCL12+) ns. **4**: (Alg:M + cells NPs-Empty vs. Alg:M + cells NPs-CXCL12) ns, (Alg:M + cells NPs-Empty vs. Alg:M + cells NPs-CXCL12+) ns, (Alg:M + cells NPs-CXCL12 vs. Alg:M + cells NPs-CXCL12+) **. The scale bar represents 2000 μm. NPs-Empty: Alginate/Chitosan-Nanoparticles unloaded; NPs-CXCL12: Alginate/Chitosan Nanoparticles loaded with CXCL12; Alg:M: Alginate:Matrigel (50:50); Alg 1%: Alginate 1% hydrogel.

We then performed the same experiment using U87-GFP GBM cells to validate whether the response of F98-mCherry GBM cells to the flow and CXCL12 gradient is consistent with that of a human GBM cell line. However, under the experimental conditions used with F98 GBM cells (0.5 μL/min flow rate with or without CXCL12 at 1600 ng/mL), few U87-GFP cells migrated within 72 h in the direction of the flow. We therefore followed the migration within 120 h and increased the concentration of CXCL12 at 2400 ng/mL (NPs-CXCL12 +) (**Fig 5**).

#### Fluorescence scans and hoechst staining

Interestingly, a strong increase in fluorescence was observed in the scans starting from 72 h in all experimental conditions notably in the presence of CXCL12, suggesting cell proliferation.

As expected, no migration front was observed in the scans for both control (NPs-Empty) and NPs-CXCL12 conditions after 72 h (**Fig 5**A). However, in the flow direction with the presence of CXCL12 (NPs-CXCL12), some individual cells were observed to migrate alone in the Alg:M section (**Fig 5**A, i). With NPs-CXCL12 at 120 h, U87-GFP cells were observed in the Alg:M section in both the directions of the flow. The cells appeared to migrate individually (**Fig 5**A, ii, iii, iv).

In contrast, with NPs-CXCL12 + (2400 ng/mL), a high fluorescence and migration front were observed in both Alg:M + cells sections in both flow direction (**Fig 5**, ii, iii, vi and iv). In the Alg 1% (w/v) section, no cell could be detected in all experimental conditions. These observations where further corroborated with the Hoechst staining (**Fig 5**B).

#### DNA quantification

The DNA quantification confirmed that adding CXCL12 enhanced the relative DNA amount (163.1% ± 2.6 and 224.4% ± 1.1 for NPs-CXCL12 and NP-CXCL12 +, respectively) compared with the control (129.8% ± 1.9; p ≤ 0.05) (**Fig 5**C). These results indicated a proliferative effect of CXCL12 on the U87-GFP GBM cells. In the control (NPs-Empty), relative DNA amount of 8.1 ± 0.1% was quantified in the Alg:M section in the direction of the flow, while no DNA was quantified in both Alg:M section against the flow and the Alg 1% (w/v) central chamber section. In the presence of NPs-CXCL12, the relative DNA amounts in the Alg:M sections in and against the direction of the flow (10.7% ± 1.4 and 32.7% ± 2.1, respectively) were significantly higher compared to NPs-Empty (p < 0.01).

With NPs-CXCL12 +, relative DNA amounts were also increased in Alg:M sections in both the flow direction (41.2% ± 2.0 and 50.1% ± 1.9, respectively) compared with the control (8.1% ± 0.2, p < 0.01). These results also indicated a strong migration compared to the first CXCL12 dose (NPs-CXCL12) (10.7% ± 1.4 and 32.7% ± 2.1, p < 0.01) (**Fig 5**C). A flow rate at 0.5 μL/min did not have a major impact on cellular proliferation, compared with CXCL12. The use of a higher concentration of CXCL12 increased the number of cells that can migrate in both directions. However, no DNA was detected in the Alg 1% (w/v) hydrogel section suggesting that the cells couldn’t reach into within 120 h.

### U87 cells migration under 3 μL/min

#### Fluorescence scans and hoechst staining

**Fig 6:**
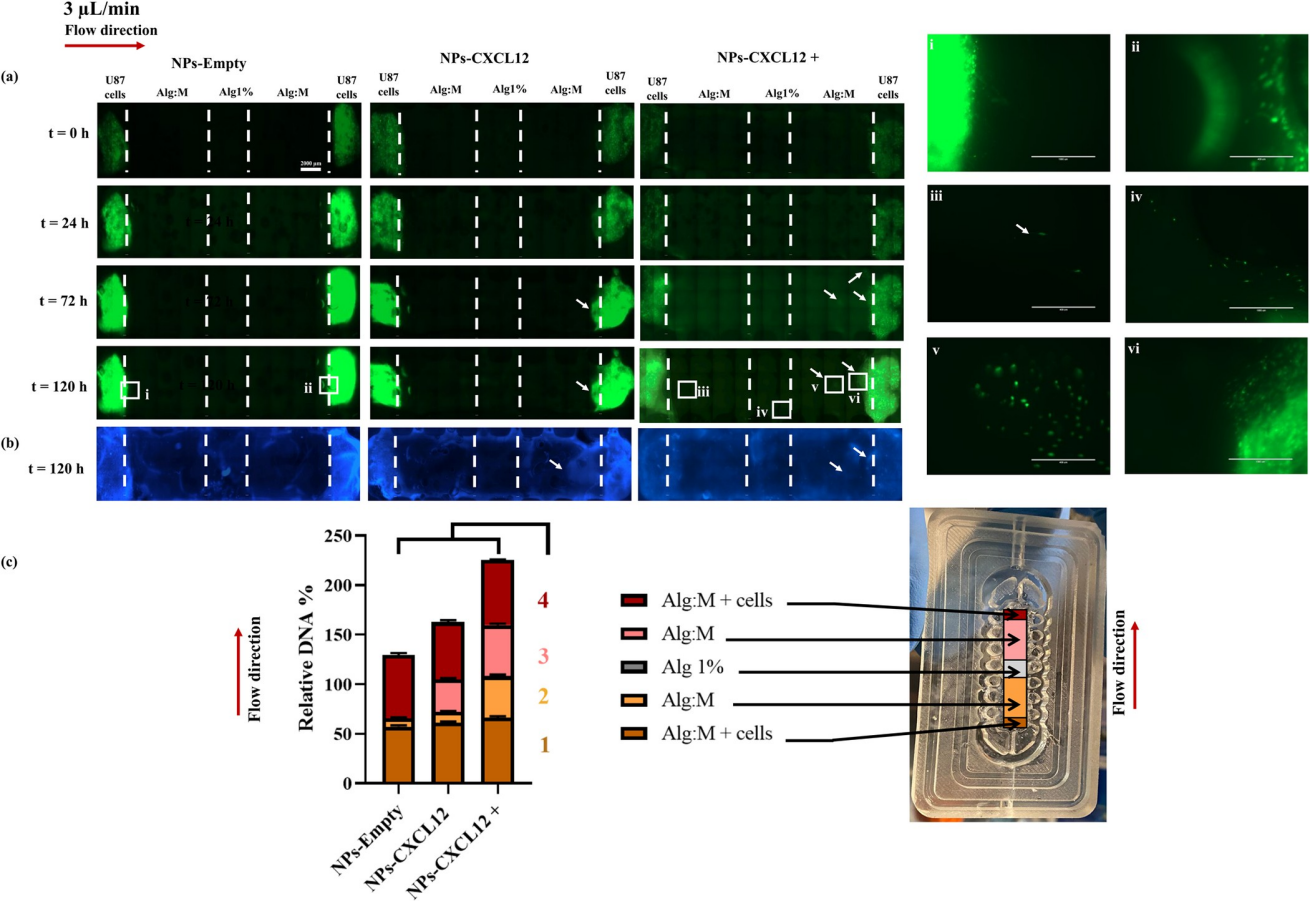
(a) Hydrogels scans of GFP U87 GBM cells migration at t = 0 h, t = 24 h and t = 120 h with and without CXCL12 at a flow rate of 3 μL/min. (b) Hoechst staining of the hydrogels at t = 120 h. (c) DNA quantification of the different hydrogel sections at the end of the experiment (N = 9, n = 3). Bars represent the mean ± SD and the squares are zoom regions. Statistical analysis was conducted using a Kruskal-Wallis test followed by Dunn’s multiple comparison test, * p *≤* 0.05, ** p *≤* 0.01, *** p *≤* 0.001. **1**: (Alg:M + cells NPs-Empty vs. Alg:M + cells NPs-CXCL12) ns, (Alg:M + cells NPs-Empty vs. Alg:M + cells NPs-CXCL12+) **, (Alg:M + cells NPs-CXCL12 vs. Alg:M + cells NPs-CXCL12+) ns. **2**: (Alg:M NPs-Empty vs. Alg:M NPs-CXCL12) ns, (Alg:M NPs-Empty vs. Alg:M NPs-CXCL12+) **, (Alg:M NPs-CXCL12 vs. Alg:M NPs-CXCL12+) ns. **3**: (Alg:M NPs-Empty vs. Alg:M NPs-CXCL12) ns, (Alg:M NPs-Empty vs. Alg:M NPs-CXCL12+) ***, (Alg:M NPs-CXCL12 vs. Alg:M NPs-CXCL12+) ns. **4**: (Alg:M + cells NPs-Empty vs. Alg:M + cells NPs-CXCL12) ns, (Alg:M + cells NPs-Empty vs. Alg:M + cells NPs-CXCL12+) ns, (Alg:M + cells NPs-CXCL12 vs. Alg:M + cells NPs-CXCL12+) **. The scale bar represents 2000 μm. NPs-Empty: Alginate/Chitosan-Nanoparticles unloaded; NPs-CXCL12: Alginate/Chitosan-Nanoparticles loaded with CXCL12; Alg:M: Alginate:Matrigel (50:50); Alg 1%: Alginate 1% hydrogel.

We therefore followed the migration within 120 h and increased the concentration of CXCL12 at 2400 ng/mL (NPs-CXCL12 +) (**Fig 6**). Interestingly, a strong increase in fluorescence was observed in the scans starting from 72 h in the control condition notably in the presence of CXCL12, suggesting cell proliferation.

As expected, no migration front was observed in the scans for the control (NPs-Empty) (**Fig 6**A, i and ii). With NPs-CXCL12 at 72 h, U87-GFP cells were observed in the Alg:M section against the direction of the flow. The cells appeared to form a slight migration front (**Fig 6**A). At flow direction of 3 μL/min, the CXC12 diffused in the direction of the flow at a proportion of 43.1% from the initial mass charged (0.33 μg of CXCL12) as described in our previous work [13] allowed the cells to debut migration.

In contrast, with NPs-CXCL12 +, a low fluorescence and migration front were observed in both Alg:M + cells sections in both flow direction (**Fig 6**A, iii and iv). In the Alg 1% (w/v) section, few cells were detected in the presence of NPs-CXCL12 + whereas there were not observed in the other experimental conditions (NPs-Empty and NPs-CXCL12). These observations where further corroborated with the Hoechst staining (**Fig 6**B).

#### DNA quantification

The DNA quantification confirmed that adding CXCL12 enhanced the relative DNA amount (162.5% ± 2.7 and 225.4% ± 2.0 for NPs-CXCL12 and NP-CXCL12 +, respectively) compared with the control (129.4% ± 1.8; p ≤ 0.05) (**Fig 6**C).

The results indicated a proliferative effect of CXCL12 on the U87-GFP GBM cells. In the control (NPs-Empty), relative DNA amount of 8.3% ± 0.8 was quantified in the Alg:M section in the direction of the flow, while no DNA was quantified in both Alg:M section against the flow and the Alg 1% (w/v) section. In the presence of NPs-CXCL12, the DNA amounts quantified in the Alg:M sections in both flow direction were 10.8% ± 1.1 and 32.8% ± 1.5, respectively, which is significantly higher compared to NPs-Empty (8.3% ± 0.8 and 0% ± 0.0, p ≤ 0.01).

With NPs-CXCL12 +, remarkable relative DNA amounts were quantified in Alg:M sections in both flow directions (42.1% ± 1.6 and 51.3% ± 1.6, respectively) compared with the control (**Fig 6**A, v). These results also indicated a strong migration compared to the first CXCL12 dose (NPs-CXCL12, 10.8% ± 1.1 and NPs-CXCL12+, 32.8% ± 1.5, p ≤ 0.01) (**Fig 6**C). However, few cells were observed in the Alg 1% (w/v) hydrogel section whereas no DNA was quantified suggesting that the amount of the existing cells was not in the detected range of DNA quantification (**Fig 6**A, iv). Contrary to what was observed at a low flow rate of 0.5 μL/min, the presence of CXCL12 (NPs-CXCL12 +) at a flow rate of 3 μL/min seems to have a major impact on cellular proliferation and migration.

### Evaluation of CXCR4 receptor mRNA and activity in F98 and U87 GBM cells

We subsequently evaluated the expression and activity of the CXCR4 receptor to ascertain whether it could account for the observed variations in cell migration and proliferation. This assessment involved quantitative analysis of CXCR4 mRNA levels, as well as functional assays to measure receptor-mediated signaling pathways. Our findings aimed to elucidate the potential role of CXCR4 in modulating these cellular processes.

#### Quantification of CXCR4 mRNA levels by qPCR

**Fig 7:**
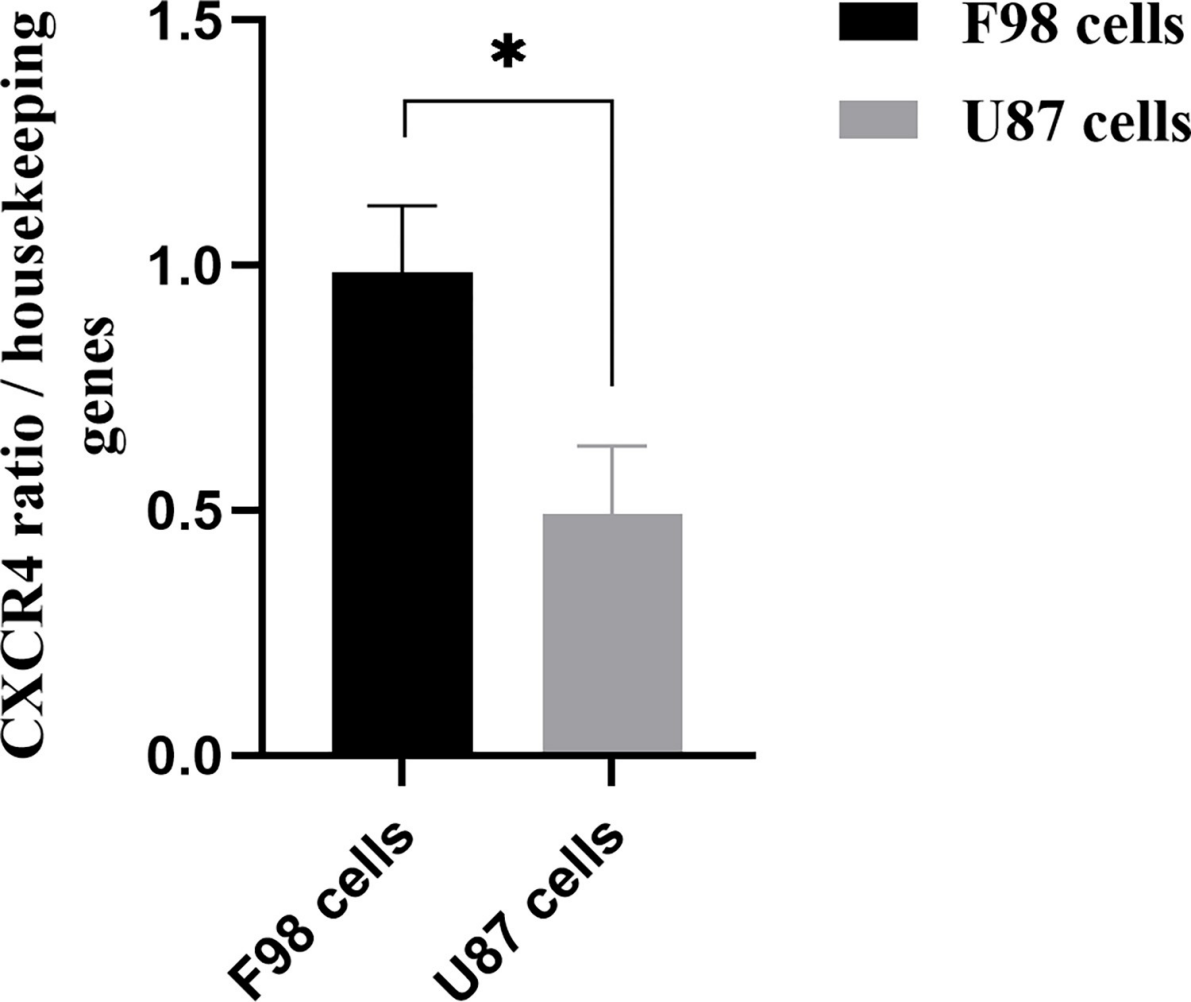
Relative CXCR4 mRNA levels in F98 and U87 cells determined by semiquantitative qPCR, *P ≥ 0.01, (N = 1, n = 3).

Both cell lines F98 and U87 express the CXCR4 mRNA as detected by semiquantitative qPCR. Comparing to the house keeping genes mentioned in Materials and Methods, the murine F98 cell line expresses twice more CXCR4 mRNA compared with the human U87 GBM line. Statistical analysis was performed using a Student t-test, *p≤0.01 (**Fig 7**).

#### CXCR4 receptor activity in F98 and U87 cell lines

Binding of CXCL12 on CXCR4 receptor results in a rapid increase in intracellular calcium concentration, which is crucial for several cellular functions, including cell migration [25–27], proliferation, as well as apoptosis [28].

**Fig 8:**
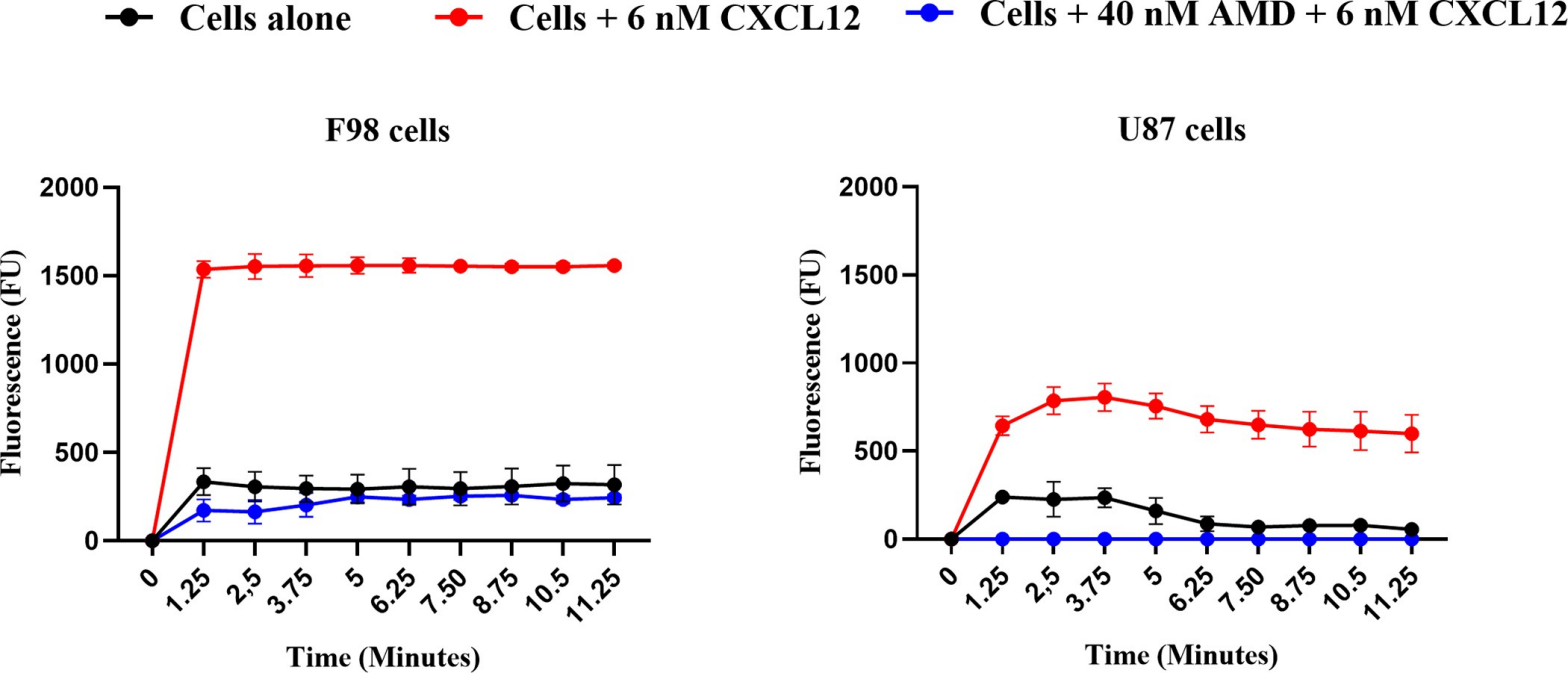
Assessment of CXCR4 receptor activity in (A) F98 and (B) U87 cells by measuring the increase in intracellular calcium flow, (N = 8; n = 3).

Activity of CXCR4 receptor in F98 and U87 cells was assessed using a calcium mobilization assay, which is based on measuring changes in intracellular calcium levels as a function of time (**Fig 8**). The activity of CXCR4 receptor expressed by the F98 and U87 cells in response to CXCL12 was first assessed. Adding CXCL12 (6 nM, 52.2 ng/mL) led to a significant increase in calcium concentration that was ~ 2.3-fold higher in F98 cells compared with U98 GBM cells (average FU measured as a function of time for F98 cells is 1397 and for U87 cells is 616). To confirm the role of CXCR4 receptor, the assay was repeated by pre-incubating the cells with the CXCR4 antagonist AMD 11070 followed by addition of CXCL12.

AMD11070 completely inhibited the activation of CXCR4 by CXCL12 in U87 cells. Regarding the F98 cells, AMD11070 did not completely prevent the increase in intracellular calcium flow, which was brought back to the level of the control without CXCL12 (P = 0.3 at 6.25 minutes) but remained at approximately 14.3% of that measured in F98 cells incubated with CXCL12 (**Fig 8**).

## Discussion

We have previously reported that the release of CXCL12 can be tuned when encapsulated into Alg/Chit-NPs and embedded into an Alg 1% (w/v) hydrogel [13]. Under dynamic indirect perfusion conditions that mimic the brain IFF, CXCL12 release and distribution inside the hydrogel are impacted by the flow rate [13]. In the present study, we analyzed further the influence of flow rate and CXCL12 on the directional distribution of cancer cells, both with and against the flow direction. To the best of our knowledge, the proposed system is the first of its kind used to elucidate the influence of flow dynamics within an *in vitro* 3D environment with the aim to assess GBM cell behaviors in response to CXCL12 chemokine gradient.

We have demonstrated that F98 express a higher level of CXCR4 mRNA than did U87 GBM cells (**Fig 7**). The activation of the CXCR4 receptor by its ligand CXCL12 increased the intracellular calcium level in both F98 and U87 GBM cells, stimulation in rat cells being 2.3-fold higher than in human cells (**Fig 8**). Previous studies have also shown that U87 cells exhibit lower CXCR4 expression than other glioma cell lines, such as the LN428, LN308 [29], and LN229 [30] resulting in lower invasion capacity.

Intracellular calcium induced by CXCR4 activation is an important factor for cell migration [31]. Unlike for F98 GBM cells, the use of AMD11070, a selective inhibitor of CXCR4 at 40 nM inhibited its activity in U87. However, it did not seem to inhibit completely the calcium elevation in F98 GBM cells suggesting other mechanisms or receptors to be implicated in the increased intracellular calcium levels in those cells.

In F98 cells, the AMD11070 prevented the increase of intracellular calcium induced by CXCL12; however, a residual raise of calcium of 14.3% persisted in the F98 cells incubated or not with the CXCL12 and the AMD11070. These results suggest that other mechanisms or receptors could also modify the level on intracellular calcium levels in the F98 cells. The receptor CXCR7 has been recently identified as a second receptor for CXCL12, and its expression has been shown to be increased in gliomas cells [32]. CXCR7 activation by CXCL12 is mediated via G-protein and β-arrestin and increases the intracellular calcium concentration [33]. Further, CXCR7 can heterodimerize with CXCR4 when activated by CXCL12 which modulates CXCR4 signaling pathways including G-protein and β-arrestin pathways like MAPK-ERK1/2, that promote cell migration [34]. The observed calcium elevation in F98 GBM cells in the presence of CXCR4 antagonist may be explained by a high expression of CXCR7 in those cells.

Further, our results showed that, under dynamic culture conditions with a flow rate of 0.5 μL/min, rat F98 GBM cells, a well-known tumor model [35], displayed migratory behavior, notably enhanced in the presence of CXCL12, both with and against the flow direction, suggesting that the chemoattractant facilitates bidirectional cell migration. Upon increasing the flow rate to 3 μL/min and using higher dose of CXCL12, a significant migration front was observed in the flow direction, with no cells migrating oppositely. The IFF allowed the diffusion of CXCL12 through the hydrogel which, in return, favored the attraction of the GBM cells following the chemokine gradient.

Our previous work in El Kheir et al., allowed to determine the amount of CXCL12 diffusing through an Alg:M hydrogel in both the flow directions at both 0.5 μL/min and 3 μL/min [13]. CXCL12 diffuses from the Alg/Chit-NPs to the Alg:M section via two steps: the molecule diffuses from the Alg/Chit-NPs to the Alg 1% hydrogel. Then, the released molecule diffuses through the Alg:M hydrogel [13]. At high flow rates, only a small amount of the molecule remained trapped, suggesting that advection is the dominant mechanism [13]. At a low flow rate (0.5 μL/min), the molecule was trapped in the Alg 1% hydrogel with a percentage of 74.9% (247 ng of CXCL12) of CXCL12 initial mass loaded (0.33 μg of CXCL12) [13]. Meanwhile, for the hydrogel in the flow direction, 15.4% (50 ng of CXCL12) diffused in the Alg:M hydrogel, whereas fewer CXCL12 molecules diffused in Alg:M against the flow (1.3% ≈ 4.3 ng of CXCL12) [13]. Increasing the flow rate to 3 μL/min allowed 43.1% (142 ng of CXCL12) of the initial mass loading to diffuse through the Alg:M section in the flow direction and 14.4% (47 ng of CXCL12) to diffuse through the Alg:M section against the flow direction [13]. The release of CXCL12 at 3 μL/min allowed a high amount of the initial mass loaded to diffuse through the Alg:M sections in both flow direction suggesting that, there is a defined flow range that promotes the distribution of the chemokine in the simulated brain parenchyma [13]. Results corroborate those of Bajetto et al., in which they found that the migration of human U87-MG GBM cells was significantly increased in a dose dependent manner by CXCL12 at a concentration varying from 1 to 50 ng/mL [36]. They have also found that the migration was still promoted with CXCL12 at 100 ng/mL compared to the control, it was lower than what had been observed at 50 ng/mL [36]. With a flow rate of 3 μL/min, the CXC12 diffused in the direction of the flow at a proportion of 43.1% (140 ng/mL ≈ 164 nM) from the initial mass charged (0.33 μg of CXCL12) as described in our previous work [13]. Which allowed the cells to debut migration. We also investigated the behavior of U87-GFP GBM to verify how IFF and CXCL12 can impact these human cells. Our results using a flow rate of 0.5 μL/min induced poor migration of U87-GFP GBM cells within 72h. This CXCL12 concentration was previously described to promote U87 GBM cell migration. Using a Transwell chemotaxis assay in a serum-free medium, Bajetto et al. found that the migration of human U87-MG GBM cells increased significantly in a dose-dependent manner with CXCL12 at concentrations ranging from 1 to 50 ng/mL. Cell migration doubled at 50 ng/mL compared to 1 ng/mL [36]. Munson et al., also found a correlation between the CXCL12–CXCR4 axis and the response to IFF on several cell lines including U87 MG (invasive rat astrocytoma C6; invasive rat astrocytoma RT2; and noninvasive rat gliosarcoma 9L) seeded in gel (0.1% hyaluronan and 0.12% collagen I) [6]. Their study established that interstitial flow (velocity of 0.72 μm/s) enhanced by two folds the invasive behavior of C6, RT2, U87MG in a CXCR4 dependent manner after 24 h, while CXCL12 at 100 nM (12 ng/mL) mainly increased the 3D invasion of RT2 and U87MG cells. They observed a positive correlation between the invasive potential of these cell lines, CXCL12 chemotactic effect and their response to IFF. Based on the results of CXCR4 expression in U87 GBM cells and the hypothesis that U87 GBM cells in our dynamic model may require more time to migrate, we not only increased the concentration of CXCL12, but also extended the migration time to 120 h. For both flow rates, increasing the dose of CXCL12 from 1600 ng/mL to 2400 ng/mL promoted the migration of U87-GFP GBM cells at 120 h in both flow directions (**Fig 5**). However, the use of high dose of the chemokine at a flow rate of 3 μL/min (NPs-CXCL12) seems to have a major proliferative impact on the cells. Meanwhile, no DNA was detected in the center of the chamber suggesting that the migration rate of U87 GBM cells under CXCL12 gradient can be further optimized. Such result may also be linked to the expression level of CXCR4 on GBM cells, which might explain the different behaviors observed between F98-mCherry cells and U87-GFP cells [37,38]. We also observed a strong proliferative effect of CXCL12 on U87 GBM cells regardless of the flow rate imposed. It has been previously reported that the CXCL12 receptor, CXCR4, stimulates a specific and significant proliferative response in GBM progenitor cells [39]. Rubin et al., used intracranial xenograft models of GBM and medulloblastoma to illustrate that the systemic administration of AMD3100 (CXCR4 antagonist) reduced proliferation and increased apoptosis in tumor cells within 48 h [40]. However, our results underscore the complex behavioral interplay between CXCL12, IFF on GBM cells migration and proliferation. Increasing the flow rate did not seem to have an impact on F98-mCherry GBM cells proliferation but it enhanced their migration. Interestingly, the migration did not increase in GFP-U87 GBM cells with the 3 μL/min flow rate. However, the use of high dose of CXCL12 (NPs-CXCL12 +) enhanced the cells migration and controlled the proliferation in those cells (**Fig 6**).

Overall, it appears that CXCL12 influences the migration and proliferation behavior of GBM cells in a concentration-dependent manner as previously described in many studies [6,36,41]. These findings suggest that the CXCR4/CXCL12 axis can differentially affect the GBM cell population leading to varied invasion behaviors in response to fluid flow. Kingsmore et al. demonstrated that the cellular response to fluid flow varies among different GBM stem cell lines (G2, G34, G62, and G528), both *in vitro* and *in vivo* [9]. In addition, CXCR4/CXCL12 chemotaxis reduced flow-stimulated invasion in some GBM cells such as G34 and G528. Those results suggest that different GBM stem cell lines respond differently to fluid flow and CXCL12, which could have implications for targeted therapies.

In addition to CXCL12, there are other chemokines that hold promise for regulating the migration of cancer cells, such as CXCL8, CXCL16, CCL2, and CXCL10. Déry et al., using other chemokines such as CXCL10, CCL11 and CCL2 investigated the migration of both F98 and human U87 MG cell lines *in vitro* [7]. They found that while CCL2 induced significant cell migration in both cell lines, the effect was slightly less prominent than that of CXCL10 [7]. However, while the *in vitro* experiments utilized the agarose drop assay, which is advantageous for studying cell migration and chemoattraction, the method had limitations in replicating the intricate 3D environment and the dynamic chemoattractant gradients observed *in vivo*. In the F98-Fischer rat model, the GlioGel, a biodegradable chemokine-loaded hydrogel, was implanted on day 3 post implantation of F98 tumor cells both within and opposite to the tumor site. The intratumoral implantation aimed to assess GlioGel’s effectiveness in limiting tumor cell migration, whereas the contralateral implantation evaluated its potential to divert tumor cells from the original site. The *in vivo* results suggested that these chemokines could limit the spread of cancer cells and potentially be used to direct migrating tumor cells. However, a significant increase in the proliferation of F98 cells was clearly observed when the GlioGel was inoculated in the tumor [7]. Although their conclusions suggested to neglect the proliferative impact on the cells due to the reducing effect on tumoral clusters [7], our findings using U87 cells *in vitro* confirmed a similar combined effect of migration and proliferation in the presence of CXCL12. Hence, we strongly believe that controlling proliferation remains a key issue that such approaches must consider.

## Conclusion

This research emphasizes the crucial role of IFF in influencing the migration patterns of GBM cells in a dynamic 3D model. Our findings reveal distinct responses of two cell lines, (rodent) F98 and (human) U87, to CXCL12 chemokines, highlighting the complexity of GBM pathobiology. We also showed that varying flow rates significantly affect brain IFF’s impact on GBM cell migration, altering the distribution of CXCL12 chemokines and subsequently influencing cell migration patterns. The correlation between chemokine gradients and cell migration patterns in our model supports the notion that targeting CXCL12 signaling could effectively mitigate GBM cell invasion. Therefore, our dynamic 3D *in vitro* model represents a significant step forward in understanding the interplay between chemokines, fluid flow, and GBM cell behavior. Considering the diverse responses displayed by the two GBM cell lines in the present study, further studies using various GBM cell lines are required to optimize the CXCL12 concentration needed to induce migration. Additionally, exploring the potential of combining different chemokines is another viable approach to overcome the current limitations such as the hypoxic microenvironment of the tumor that influence the chemokines expression in GBM cells. The model developed may be used under hypoxic conditions for future investigations in this regard.

## Supporting information

S1 Raw data ElKheir RawData. (ZIP)
